# Techniques for subretinal injections in animals

**DOI:** 10.1111/vop.13219

**Published:** 2024-05-03

**Authors:** Ryan F. Boyd, Simon M. Petersen‐Jones

**Affiliations:** ^1^ Charles River Laboratories Mattawan Michigan USA; ^2^ Department of Small Animal Clinical Sciences Michigan State University East Lansing Michigan USA

**Keywords:** animal model, retina, subretinal injection

## Abstract

Subretinal injections are not commonly performed during clinical treatment of animals but are frequently used in laboratory animal models to assess therapeutic efficacy and safety of gene and cell therapy products. Veterinary ophthalmologists are often employed to perform the injections in the laboratory animal setting, due to knowledge of comparative ocular anatomy between species and familiarity with operating on non‐human eyes. Understanding the different approaches used for subretinal injection in each species and potential complications that may be encountered is vital to achieving successful and reproducible results. This manuscript provides a summary of different approaches to subretinal injections in the most common animal model species, along with information from published literature and experience of the authors to educate novice or experienced surgeons tasked with performing these injections for the first time.

## 1 PURPOSE

This paper provides a review of approaches for subretinal injection in a variety of companion and experimental animal species.

## 2 INTRODUCTION

Subretinal injections are used primarily for introduction of therapeutic products into the subretinal space. They have been performed in experimental animals for over 40 years for different purposes including inducing experimental retinal detachments and development of cell and gene therapy for retinal disease. Therapeutic subretinal injections in human ophthalmology have also been used for many years to treat submacular hemorrhage associated with age‐related macular degeneration by introducing tPA.^1^ More recently, subretinal injections were used in clinical trials for gene therapy for retinal dystrophies including Leber Congenital Amaurosis (LCA) due to biallelic mutations in the *RPE65* gene.^2^, ^3^ The outcome of one of these clinical trials for gene augmentation therapy for the treatment of LCA‐*RPE65* is the first FDA approved gene therapy product, voretigene neparvovec‐rzyl (Luxturna).^4^ There are several other ongoing clinical trials utilizing subretinal injection for the treatment of human retinal dystrophies (see Cheng and Punzo^5^). Currently, the most efficient way of delivering a gene therapy product to the retinal pigment epithelium or photoreceptors is by subretinal injection.

Treatments delivered via subretinal injection include viral or non‐viral vectors for the introduction of therapeutic genes to the outer retina (RPE and photoreceptors). It may also involve the infusions of cells, such as retinal pigment epithelium (either harvested from a donor or grown “in the dish”) or other transplanted cells such as retinal progenitor cells. Although these treatments may not be available to companion animals as an individual animal therapeutic in the near future, a significant amount of preclinical efficacy and safety assessment is being performed in animal models to develop these treatments for humans. Few human vitreoretinal surgeons have experience performing these injections in animal models; therefore, veterinary ophthalmologists are often solicited to perform the injections.

Techniques for subretinal injection have evolved over the last 30 years, with a goal of creating a more controlled and less invasive procedure for initiating retinal detachment and slowly filling the subretinal bleb while monitoring for complications. A manual infusion technique in which the plunger of the injection syringe was removed (leaving the rubber stopper) and attached to a second syringe operated by an assistant that would drive the rubber stopper for controlled infusion was described by Wallace and Vander.^6^ This was further modified to replace the assistant driving the rate of injection with the viscous fluid infusion (VFI) function of a vitrectomy machine.^7^ The VFI allows the surgeon to control infusion with the foot pedal and a maximum infusion pressure can be set for when the pedal is maximally depressed. Adapters to connect the injection syringe to the VFI system are now commercially available and being utilized in human patients (https://medone.com/microdose‐injection‐device/). Ophthalmic operating microscopes with OCT attached are now available and are being used by vitreoretinal surgeons to monitor the accurate placement of the subretinal injection.^8^ A detailed step‐by‐step procedure for subretinal injection for gene therapy in humans was recently published and serves as an excellent reference for a novice surgeon.^9^ This technique describes use of a saline “pre‐bleb” to mitigate reflux into the vitreous, where the initial retinal detachment is created using a small amount of balanced salt solution, followed by infusion of the intended therapeutic solution. Another recent article summarizes the standard and alternative subretinal injection techniques used in humans, along with a review of potential complications, comparison to intravitreal and suprachoroidal delivery, and a brief description of different approaches used in animal models.^10^

## 3 APPROACHES TO SUBRETINAL INJECTION

### 3.1 Open sky approach

Early approaches were an “open sky” approach. This was used in an experimental cat model of induced retinal detachment. At a prior surgery, lens removal was performed after which the eye was allowed to recover. After recovery, the injection technique was performed that involved a vitrectomy including a retinotomy that was created with the vitreous cutter and then infusion into the subretinal space using a curved needle.^11^ A similar open sky approach was used for transplantation of RPE in non‐human primates.^12^ This rather invasive approach currently does not appear to be commonly used and will not be discussed further.

### 3.2 Trans‐scleral approach

Approaches through the sclera, choroid and RPE to access the subretinal space have been predominantly used in rodents. Early descriptions were of a technique that gained access to the sclera at the posterior of the globe via entry through an incision through the upper lid and reflection of the dorsal rectus muscle.^13^ Lazar and del Cerro^14^ reported a less invasive trans‐scleral approach in which a microneedle with an outer plastic sleeve positioned to limit depth of penetration of the needle was introduced through the sclera and choroid to access the subretinal space. This technique was also reported in dogs.^15^ Currently, the trans‐scleral approach is not routinely used in humans, but medical devices manufactured specifically for this purpose are in development. The use of one device has been reported in pigs and dogs.^16^, ^17^

**FIGURE 1:**
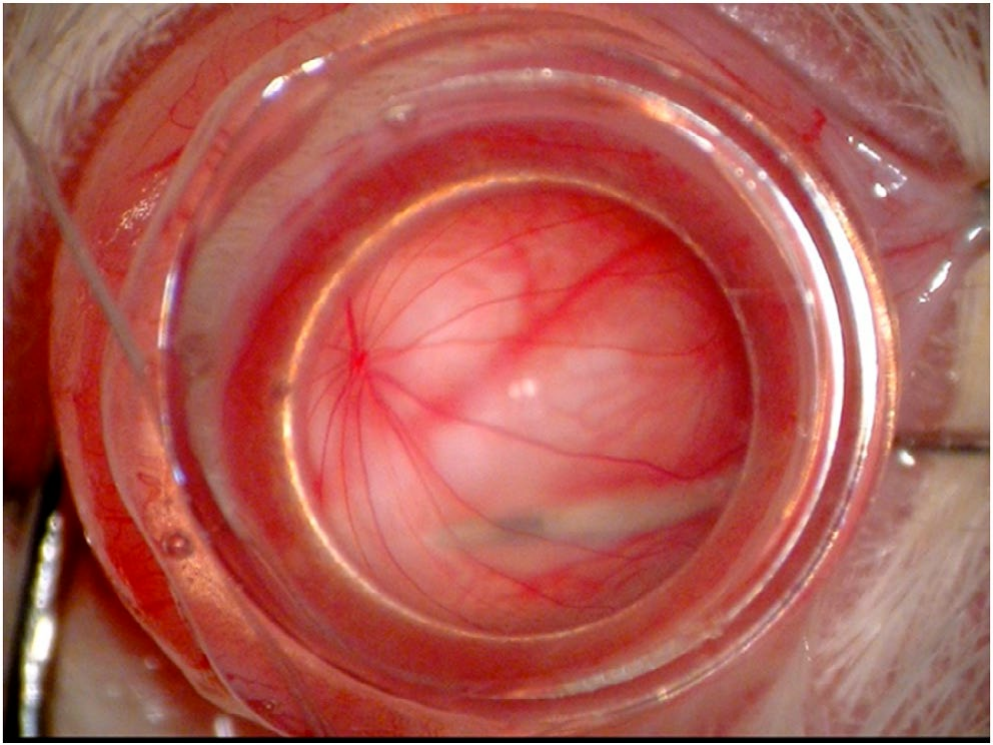
Rodent intravitreal contact lens. A custom rodent intravitreal lens has been placed on the cornea using methylcellulose coupling gel, facilitating clear visualization of the retina and needle approach for subretinal injection in this albino rat.

In rodents, the trans‐scleral approach is often used due to small globe size and ability to proptose the eye for access to the posterior globe. This procedure can be performed without direct visualization in neonatal rodents.^18^, ^19^ or under direct observation of the posterior pole of the eye through an operating microscope.^20^ A small circular microscope coverslip or specific rodent contact lens coupled with a viscous gel can be used to allow the fundus to be visualized as the needle is advanced into the subretinal space (Figure 1, https://ocuscience.us/collections/products/products/rodent‐intravitreal‐lens‐mouse). Posterior trans‐scleral approaches do not disturb the clear ocular media and avoid damaging the lens, making post‐dose ocular examinations and diagnostic procedures more straightforward. Disadvantages of the trans‐scleral approach include risk of retrobulbar reflux of injected solution, a more limited region of treatment compared to some transvitreal methods, and risk of choroidal hemorrhage. Retrobulbar reflux is likely the result of IOP elevation following injection, this can be mitigated by penetration of the cornea with a 30–32 g needle to lower IOP following needle placement in the subretinal space but prior to injection of solution.^21^

### 3.3 Transvitreal approaches

Standard subretinal injection techniques in large animals mimic the procedure used in human patients,^9^ utilizing a transvitreal approach. In humans, vitrectomy is part of the standard approach for transvitreal subretinal injection due to perceived risk of postoperative proliferative vitreoretinopathy (PVR). The main advantages of vitrectomy include the ability to control IOP during the injection and perform a fluid‐air exchange following the injection to remove any of the injected solution that may have refluxed into the vitreous chamber during subretinal injection.^22^ Complications associated with vitrectomy include increased postoperative inflammation, prolonged hypotony, delayed retinal reattachment and eventual cataract formation. In large animal models, these complications can be more prevalent due to the inability to restrict activity levels postoperatively. Therefore, vitrectomy is infrequently part of the standard subretinal injection technique in animals. In the combined experience of the authors, they have not observed a case of PVR formation after subretinal injection of a non‐cellular material into a large animal eye where vitrectomy was not performed. Stem cell injection has the potential to cause PVR with or without vitrectomy.

In rodents, transvitreal injections can be performed by introducing the injection cannula through a stab incision made with a hypodermic needle through the cornea^23^, ^24^ or posterior to the limbus.^25^, ^26^ These approaches have different advantages and disadvantages. Transcorneal approaches are more likely to accomplish a larger subretinal bleb due to hypotony induced by the stab incision through the cornea, resulting in greater retinal surface area covered by the injection, with 50%–100% of the retina often detached.^23^, ^24^ This can be a greater advantage for efficacy studies in transgenic rodents where rescue of an ERG phenotype is important. Pars plana approaches typically result in smaller subretinal blebs within the quadrant of the injection. Disadvantages to the transvitreal approaches include lens opacities resulting from needle contact with the large rodent lens, which can impact visualization of the fundus during follow‐up examinations or OCT imaging and inhibit accurate ERG recordings. This is particularly prevalent in transcorneal approaches, and less of a concern when an experienced surgeon performs a transvitreal injection via an approach just posterior to the limbus. Retinal or vitreal hemorrhage and vitreal reflux of injected solution are also common complications when utilizing a transvitreal approach. A recent publication reported a significant amount of training is required to achieve a 95% success rate utilizing a transvitreal approach in mice, finding the surgeon needs to perform approximately 364 cases to achieve that standard.^27^

## 4 EQUIPMENT

### 4.1 Surgical microscope

An ophthalmic surgical operating microscope with co‐axial illumination and an assistant's eyepieces are needed.

### 4.2 Visualization of posterior segment

A method to visualize the posterior segment through the operating microscope is needed. This can range from a simple small glass coverslip or contact vitrectomy lens (e.g., Volk VFD10 (https://www.volk.com/products/volk‐1‐single‐use‐flat‐lens) or similar disposable lens) that are coupled with sterile viscous gel, to an irrigating vitrectomy lens that is held in position by an assistant, to a non‐contact system such as the Oculus Biom (https://www.oculussurgical.com/biom‐ready). The choice depends on the species being injected and the surgeon's preference.

### 4.3 Vitrectomy machine

This is required if a vitrectomy is planned or if the viscous fluid infusion (VFI) pump is to be used to control the rate of injection. It is not required for subretinal injections in rodents or in larger animals if a vitrectomy is not to be performed and the VFI feature not utilized.

### 4.4 Subretinal cannula

The choice of injection cannula or needle will be dependent on the approach being used (Trans‐scleral vs. transvitreal) and surgeon's choice. There are injectors with a retractable narrow‐gauge cannula such as the Retinoject that was popular but is no longer available^28^ and a cannula made by DORC (https://dorcglobal.com/product/extendible‐41g‐subretinal‐injection‐needle‐23‐gauge‐06‐mm) which has a 23‐gauge blunt needle from which a 41 gauge polytetrafluoroethylene cannula can be extended for the actual injection. Other injection needles with a flexible fixed cannula extended from a metal blunt needle are also available (e.g., MedOne PolyTip Cannula 25 g/38 g; https://medone.com/sub‐retinal‐cannulas/). Another retractable cannula option has been produced by Vortex Surgical, which uses a 28 g straight or curved self‐penetrating outer needle with a 41 g beveled injection needle (https://www.vortexsurgical.com/irrigationinjection).

## 5 SPECIES‐SPECIFIC APPROACHES

### 5.1 Rats and mice

For both transvitreal and trans‐scleral approaches in rodents, injectable (ketamine/zylazine or ketamine/dexmedetomidine) or inhalant anesthetics are required for immobilization. Pupils should be dilated with 0.5%–1% tropicamide and 2.5% phenylephrine. The animal should remain on a heating pad as much as possible during anesthetic induction and the injection procedure, and corneal lubricant should be applied at the time of anesthetic induction. Mice are particularly prone to development of reversible anesthesia‐induced cataracts, which can preclude fundus visualization making injection more difficult or impossible. These transient cataracts are thought to arise from multiple factors including corneal drying and hypothermia.^29^, ^30^ In the authors' experience, application of a hypromellose‐based or similar gel lubricant under a basic rodent contact lens (https://ocuscience.us/collections/products/products/mini‐contact‐lens) significantly delays onset of cataract formation. If cataracts are observed, they will generally resolve within 1 h of anesthesia recovery.

**FIGURE 2:**
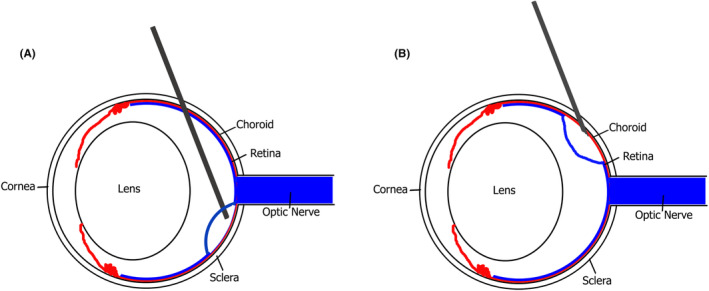
Approaches for subretinal injection. Schematic of a rodent eye illustrating the needle path taken for transvitreal (A) and trans‐scleral (B) approaches. Note that a sharp beveled needle is required for a standard trans‐scleral approach to facilitate scleral and choroidal dissection, while a blunt needle may be used for the transvitreal approach following creation of a pilot sclerotomy hole.

For globe exposure, the eye can be manually proptosed in mice by applying pressure to the eyelids, while in rats a wire eyelid speculum placed on the eyelids or behind the globe will significantly aid the injection. A custom rodent lens (https://ocuscience.us/collections/products/products/rodent‐intravitreal‐lens‐mouse) or microscope coverslip should be placed on the cornea for direct visualization of the fundus. A stab incision can be made using a 30–32 g needle for anterior approaches, followed by insertion of a blunt or angled (up to 45 degrees) 32–34 g needle through the vitreous and onto the retina (Figure 2A). For a posterior trans‐scleral approach, a sharp beveled 32–34 g needle (https://www.hamiltoncompany.com/laboratory‐products/needles) is inserted slowly through the sclera and choroid until the entire bevel is through the choroid, adjusting the angle so as needed to keep the needle tip under the retina (Figure 2B, Video 1). Once the needle tip is in place for both approaches, the solution is slowly injected, and bleb formation should be immediately visualized. Injection volumes in rodents range from 0.5 to 2 μL in mice up to 5–10 μL in rats.

**VIDEO 1:** Trans‐scleral subretinal injection in the rat. A loop of flexible Kirshner wire has been placed behind the globe for increased exposure, and a rodent intravitreal injection contact lens has been placed on the cornea for visualization of the fundus. Globe fixation is achieved using 0.12 mm Colibri forceps, and a 32 g Hamilton needle is gently advanced behind the globe equator at a shallow angle. The needle is inserted through the sclera and choroid until the entire bevel of the needle is visualized within the subretinal space, as evidenced by increased clarity of the needle tip and bevel as it passes through the choroid. 5 μL of diluted fluorescein solution is slowly injected into the subretinal space, with filling occurring in a semicircular pattern indicating the injection is not intravitreal. At the conclusion of the injection as the needle is withdrawn, return of retinal vascularization over the subretinal bleb is briefly visualized.

The most common complications following subretinal injection in rodents include retinal or choroidal hemorrhage, lens opacification, and persistent retinal detachment. Prevalence of the complications vary based on the specific approach used, with anterior transvitreal approaches more likely to cause cataract formation and retinal hemorrhage, and posterior trans‐scleral approaches more likely to cause choroidal hemorrhage. Persistent retinal detachment is more often observed following procedures that induce hypotony as part of the injection procedure. With all approaches, surgeon experience is the biggest factor affecting rate of intraoperative or postoperative complications, emphasizing the need for sufficient practice to achieve a desirable outcome.

### 5.2 Large animals

**FIGURE 3:**
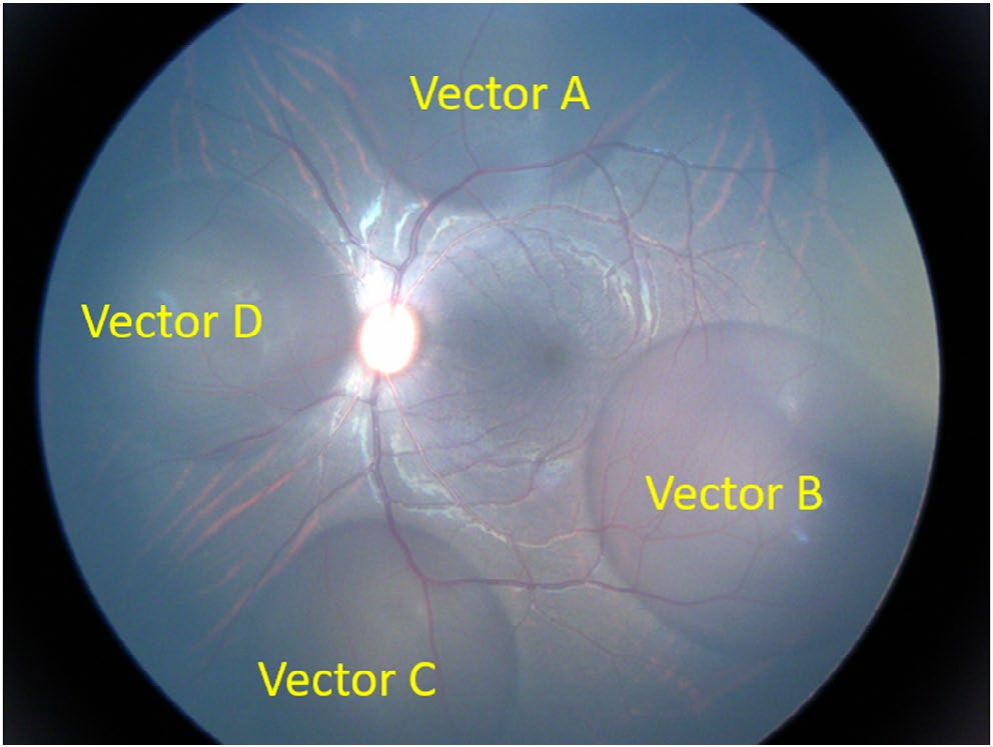
Multiple subretinal blebs in a non‐human primate. The low lens:vitreous volume ratio in primates enables the surgeon to easily access all quadrants of the retina from a single approach. In this case, four 30 μL subretinal blebs were created to compare efficiency of four different viral vector solutions in a single eye, reducing the number of animals required for the investigation.

When performing subretinal injections in large animal models, differences in globe and lens size and retinal anatomy will influence placement of the subretinal injection. Non‐human primates have a fovea and macula, and the very low lens/globe ratio allows placement of the injection within any of the four quadrants. This permits creation of up to four separate injection blebs to compare different therapies in a single eye (Figure 3). Other large animal species have higher lens:globe ratios that limit instrument mobility within the vitreous, and differences in location of the cone‐rich visual streak and area centralis between species may influence where the injection needs to occur.^31^ Another factor that influences instrument mobility within the vitreous is the location of the pars plana in each species. For placement of scleral ports through the pars plana, measurements posterior to the limbus should be 1.5–2.0 mm in rabbits, 3.0–4.0 mm in non‐human primates and pigs, and 5.0–6.0 mm in fully grown dogs and cats. In juveniles, the scleral ports are placed proportionally closer to the limbus.

For standard subretinal injections in large animal species, general anesthesia is required using inhalant anesthetics. Pupils should be dilated with 1% tropicamide and 10% phenylephrine, application of 1% atropine the day prior or the morning of injection can help ensure adequate mydriasis. The eyelid margins are generally prepped with 5% betadine solution, while the ocular surface is prepped with topical anesthetic and diluted (0.1%–1%) betadine:saline solution.^32^ An additional application of 5% betadine solution is applied to conjunctiva over the site of scleral port placement. In most species, lateral canthotomy is not required for subretinal injections without vitrectomy, a wire eyelid speculum provides sufficient exposure to place two scleral ports. Scleral ports are available in sizes ranging from 20 to 27 g, the authors prefer use of 25 g valved ports which prevent the need for postoperative closure of sclerotomies and allow the use of instruments rigid enough to allow manipulation of eye position. 27 g instruments are more prone to bending during the procedure, and larger ports will encourage vitreous leakage from the sclerotomies and persistent postoperative hypotony. There are many options for subretinal cannulas, some manufacturers only produce cannulas in one diameter size, so this needs to be considered when selecting cannulas and scleral ports. Cannulas come in fixed and extendable options; in both cases the tip of the cannula can be trimmed at an angle of 30–45 degrees to ease bleb induction. 23 and 25 g ports can be placed through the conjunctiva and sclera in an offset manner, using a two‐plane approach for a suture‐less procedure.

The animal's head should be positioned so that the cornea is horizontally positioned, conjunctival stay sutures can be useful for positioning. The conjunctiva and episcleral tissues can be reflected at the site for scleral port placement. Wet‐field cautery can reduce the risk of bleeding from scleral vessels. Care should be taken to avoid scleral and episcleral vessels. Two scleral ports are placed to facilitate use of a subretinal injection cannula and an endoilluminator for visualization. Careful attention must be paid to the angle of the scleral ports and intraocular instruments during insertion; they must be directed toward the posterior pole to avoid contacting the lens (Video 2). In dogs and cats, presence of the reflective tapetum means that an endoilluminator is not essential and the microscope light can be adequate. If vitrectomy is not being performed, aqueous paracentesis can be performed to reduce IOP and facilitate initiation of retinal detachment. Vitrectomy requires placement of a third scleral port for infusion of balanced salt solution, placement of this third port may require a lateral canthotomy to increase exposure. A vitrectomy lens or other viewing system is placed to perform the vitrectomy or to start the approach for subretinal injection. Following introduction of the cannula and illuminator through the scleral ports, the surgeon should center the microscope view on the intended dosing site and increase magnification, with fine focus on the nerve fiber layer of the retina. If an extendable injection cannula is being used, it should be extended within the mid‐vitreous before the approach to the retina is performed. Once the intended retinotomy site is in focus, the cannula tip can be advanced carefully through the posterior vitreous until it contacts the inner retinal surface. In animals with a thick vitreous, bending of the flexible cannula can occur and care needs to be taken to approach the retinal surface at the desired angle to start the detachment. With sufficient magnification and illumination, a beveled cannula tip can be visualized dissecting through the retina, but most often a slight indentation of the retina is the best visual indication of a properly positioned cannula. Choice of retinotomy site is dependent on the intended treatment outcome, but the cannula is most often placed adjacent to a retinal vessel, which seems to provide stability. Injection of solution is then initiated by slow depression of the vitrectomy machine foot pedal, with pressure increased until bleb induction can be visualized. The authors prefer a maximum infusion pressure setting of 14 psi for most large animal species (but higher in cats may be needed), with 8–10 psi often required for bleb initiation. If bleb initiation cannot be achieved, the max infusion setting can be increased, but care must be taken to not use too much pressure, this has been associated with negative postoperative effects.^33^ The analogy has been made that this is like blowing up a balloon; higher pressure is needed initially to start the expansion but less pressure is then needed once the expansion starts. Once bleb induction has been visualized, the cannula tip should remain within the retinotomy but retracted slightly to avoid RPE damage, and infusion should continue at a very slow rate, generally requiring pressure of 6–8 psi. An assistant monitors the amount of solution being injected, verbally notifying the surgeon for each 10 μL of solution administered, and the bleb is filled until the targeted dose volume is achieved. The surgeon should constantly observe cannula tip placement to avoid enlargement of the retinotomy, and monitor for development of other complications like hemorrhage, RPE detachment, or formation of a foveal hole in non‐human primates. Once the injection is complete, the instruments are removed from the eye and scleral ports are removed, and the animal should receive local and systemic anti‐inflammatory inflammatory and antibiotic treatment for up to 1 week postoperatively. Local prophylactic treatment options include subconjunctival injection triamcinolone or methylprednisolone and cefazolin or gentamicin. Systemic prophylaxis typically involves a course of corticosteroid or non‐corticosteroid anti‐inflammatory treatment, as well as oral or injectable antibiotics. Species appropriate analgesic drugs are provided to give continued analgesia.

**VIDEO 2:** Approach for subretinal injection in large animals (minipig). A Castroviejo caliper is used to make depressions within the sclera 3 mm posterior to the limbus, approximately three clock hours apart. Bipolar point cautery is briefly applied to mitigate hemorrhage during placement of the scleral ports. 5% betadine solution is applied to the conjunctiva over the sites for scleral port placement and aqueous paracentesis. The conjunctiva is retracted from the marked spot to provide an offset conjunctivotomy/sclerotomy, and each trocar tip is anchored into the sclera at the marked spot first at a shallow angle, then the angle is steepened as the trocar is inserted through the sclera aiming for the posterior pole to avoid contact with the lens. Once both scleral ports are in place, aqueous paracentesis is performed to induce hypotony and ease initiation of retinal detachment during subretinal injection.

**FIGURE 4:**
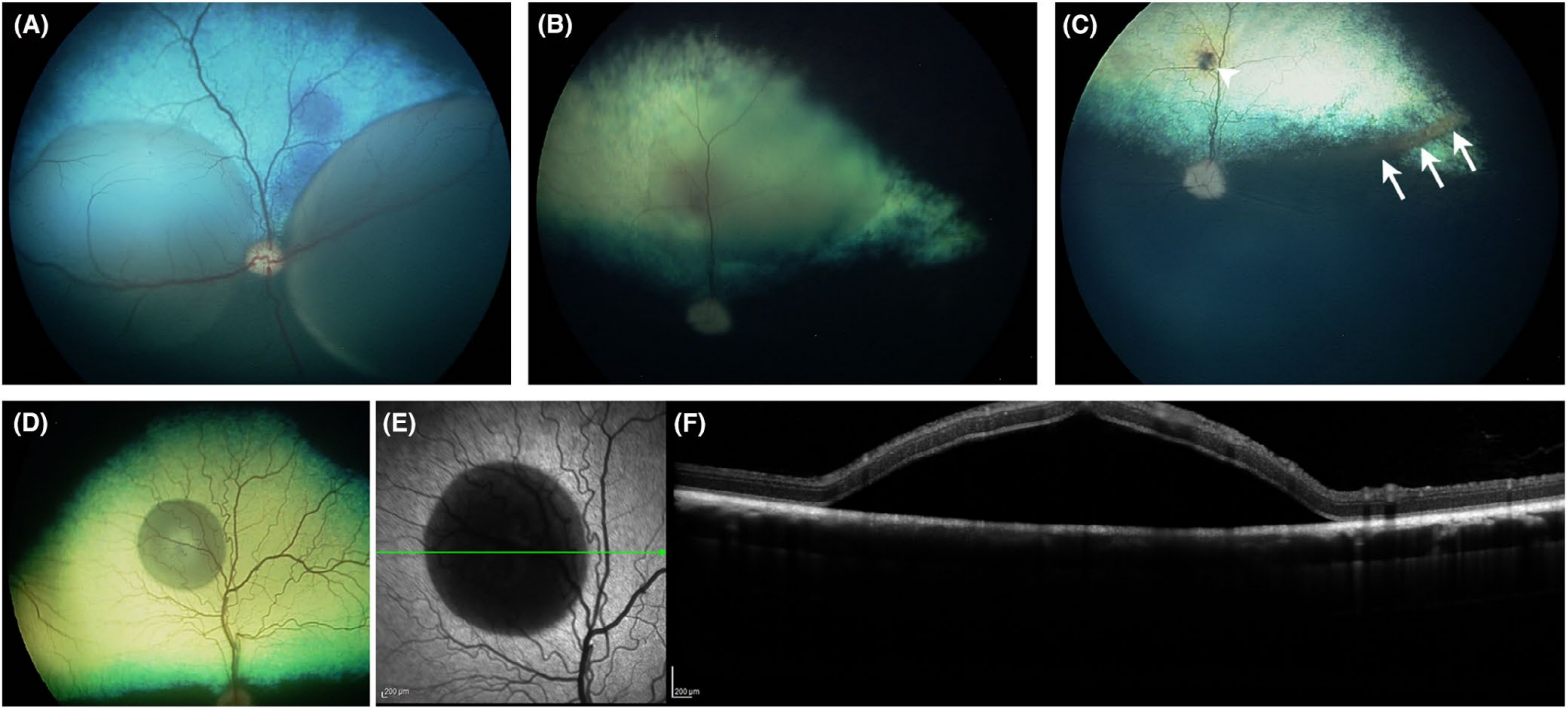
Subretinal injections in dogs. (A) Fundus image immediately after creating two subretinal blebs in a 3‐month‐old dog with normal retinal thickness. (B) Subretinal injection in a dog with moderate degree of retinal thinning due to PRA. The bleb is flatter than those created in the dog in (A). (C) Same dog as in (B) 24 h after injection. The bleb has flattened and there is some subretinal debris at the ventral edge of the bleb (indicated by arrows). The retinotomy site is indicated by the arrowhead. (D–F) Single small subretinal bleb in a normal dog. (D) Color RetCam image. (E) Confocal scanning laser ophthalmoscope (cSLO) infrared (IR) image. The green line indicates the plane of the spectral domain optical coherence tomography (SD‐OCT) cross‐sectional image shown in (F).

**FIGURE 5:**
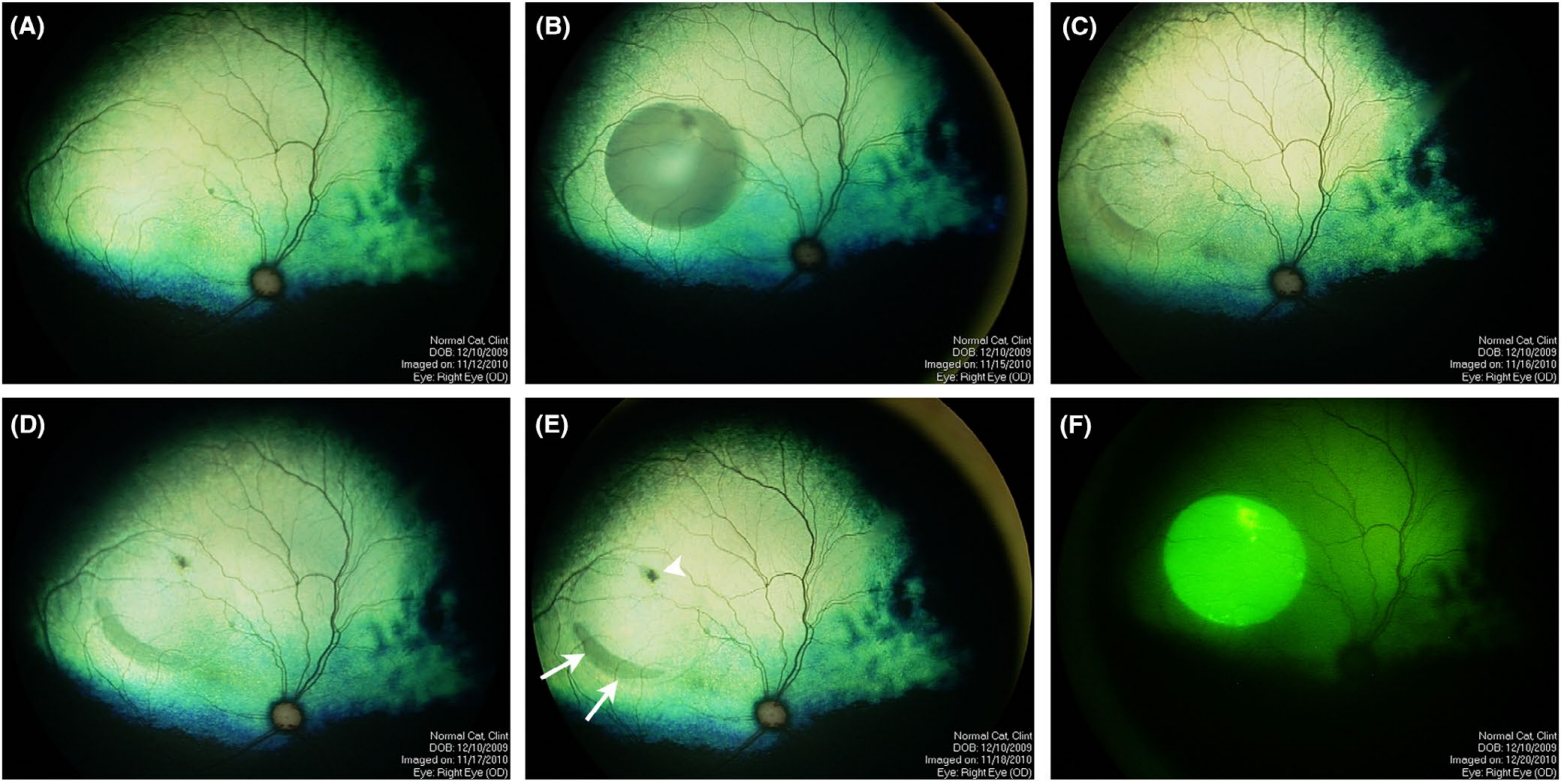
Subretinal injections in a cat. Cat was injected with an adeno‐associated viral vector delivering a green fluorescent protein marker gene. (A) Fundus prior to injection. (B) Image immediately post‐injection. (C–E) Fundus images 1‐, 2‐ and 3‐day post‐injection. These show that the subretinal fluid is cleared rapidly allowing retinal reattachment. The retinotomy site is indicated by the arrowhead in (E). Note also the subretinal debris accumulated at the ventral (dependent) portion of the bleb indicated by arrows in (E). (F) Fluorescent imaging showing GFP expression of the transduced retinal cells in the region of the bleb.

Complications of subretinal injections in large animals are similar to those encountered in rodents, with retinal or choroidal hemorrhage at the retinotomy site being the most common intraoperative observation. Postoperatively, one can expect to observe transient accumulation of subretinal debris within the gravity‐dependent portions of the bleb, which likely represents cellular material shed from the photoreceptors and RPE during the induction of retinal detachment (Figures 4 and 5).^34^, ^35^, ^36^ This will often resolve by 8 weeks post‐injection. The examiner will also frequently observe a reduction in RPE pigmentation within the bleb margins, related to irregular RPE pigment distribution following retinal detachment and reattachment.^35^, ^36^ Species‐specific considerations and complications are listed below.

#### 5.2.1 Dogs

The sclera can be tough to penetrate, a 25 g hypodermic needle may be utilized to make the initial penetration making it easier to then introduce the port. Once the port(s) are in place an aqueous paracentesis can be performed to make developing the bleb easier. The tapetum in dogs (and cats) often provides enough light reflection from the surgical microscope to enable subretinal dosing over the tapetal retina without endoillumination and only having a single scleral port for the injection cannula. The use of a second port with an endoilluminator can be useful and also helps in the positioning of the globe. When pressing the cannula tip on the retinal surface care should be taken to not press too hard as this can lead to intratapetal injections which appear as a rapid outward spread of altered tapetal color. The degree of pre‐existing retinal degeneration can influence the shape of the bleb that forms. In the authors' experience a thinned retina, for example due to an inherited retinal degeneration, will develop a flatter and therefore wider detachment than typically achieved in a retina of normal thickness. Standard subretinal injection volumes in dogs are up to 100 μL for a single bleb, with as much as 150 μL possible in a normal retina without significant complications. Figure 4 shows subretinal injections in dogs. Video 3 shows subretinal injections in a dog.

**VIDEO 3:** Subretinal injections in a dog. A single port approach was used relying on light from the operating microscope with a Volk contact vitrectomy lens on the cornea to allow visualization of the posterior segment. A MedOne PolyTip Cannula 25 g/38 g with an obliquely cut tip is introduced through the previously placed 25 g valved port positioned through pars plana. The cannula is advanced to the retinal surface and the first bleb created. The cannula is then repositioned and a second bleb created.

#### 5.2.2 Cats

Placement of scleral ports should be considered carefully to avoid the large scleral vascular plexus often present. Reflection of the conjunctiva and wet‐field cautery at the sclerotomy site prior to placement avoids most bleeding. As with the dog, aqueous paracentesis following scleral port placement helps provide “space” for the injected fluid. In cats, a little more fluid pressure is required to start the detachment than is needed for subretinal injections in dogs. The retina does not always reattach evenly if a large bleb is created; some retinal folds may result. It is better to create multiple blebs if treatment of a large retinal area is desired. Standard subretinal injection volumes in cats are up to 100 μL for a single bleb; however, the authors prefer to induce multiple blebs of up to 50 μL. Figure 5 shows subretinal injections in cats. Video 4 shows a subretinal injection in a cat.

**VIDEO 4:** Subretinal injection in a cat. A single port approach was used relying on light from the operating microscope with a Volk contact vitrectomy lens on the cornea to allow visualization of the posterior segment. A MedOne PolyTip Cannula 25 g/38 g with an obliquely cut tip is introduced through the previously placed 25 g valved port positioned through pars plana. The cannula is advanced to the retinal surface and the bleb created.

#### 5.2.3 Rabbits

**FIGURE 6:**
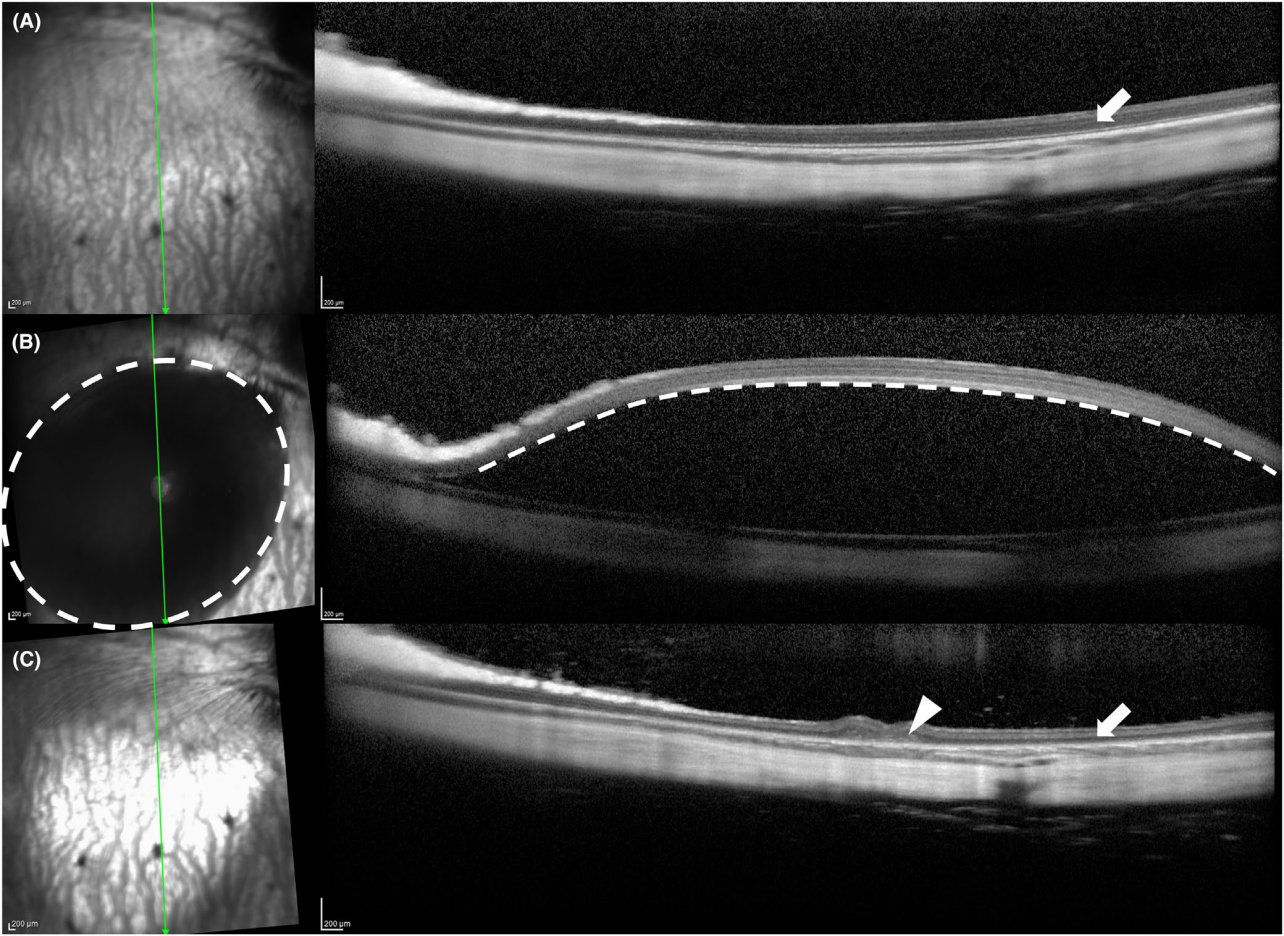
Retinal degeneration following subretinal injection in a rabbit. The thin retina of the rabbit is not conducive to recovery from routine subretinal injection, retinal degeneration is often observed. (A) Predose IR cSLO, (left panel) and SD‐OCT (right panel) images showing normal outer nuclear layer thickness in the rabbit (white arrow, hyporeflective layer). (B) Immediate post‐dose images following 70 μL subretinal injection, showing a subretinal bleb on cSLO with separation of the outer retina from the underlying RPE and choroid on SD‐OCT (white dashed lines). (C) Images taken 30 days post‐dose demonstrate diffuse outer nuclear layer thinning (white arrow) with central outer retinal folds (white arrowhead) within the subretinal dosing site.

**FIGURE 7:**
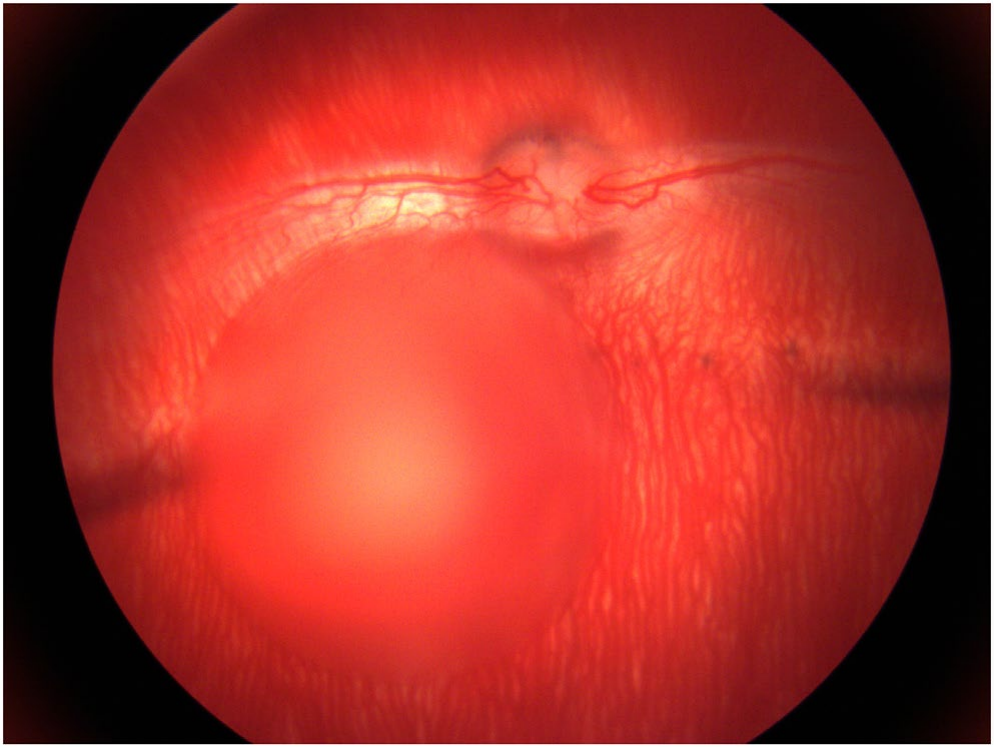
Subretinal injection in a rabbit. If subretinal injection is performed in the rabbit, the subretinal cannula should be placed along the inferior margin of the myelinated medullary nerve fibers, which most often leads to a subretinal bleb that expands in an inferior direction to involve the visual streak, as shown in this photograph taken immediately following injection.

Rabbits are not generally considered an acceptable model for subretinal injection due to high prevalence of retinal degeneration following subretinal injection of inert solutions like buffered saline.^34^, ^37^ This observation has been confirmed by the authors (Figure 6) and is likely a factor of the less vascular nature of the rabbit retina, as well as the lower normal retinal thickness. If the rabbit is selected as the model used for subretinal injection, special care must be taken to avoid lens damage during the approach from the pars plana, which is only 1.5–2 mm posterior to the limbus. The horizontal myelinated retinal vascular arcades are relatively easy to access via a superior approach, and placement of the retinotomy along the inferior margin of the myelinated nerve fibers seems to provide more stability and a higher rate of success (Figure 7). Volumes of 50–70 μL have been injected as single blebs by the authors with a high rate of retinal degeneration occurring. Lower injection volumes resulting in less stretching of the retina and faster retinal reattachment may yield better results.

#### 5.2.4 Pigs

**FIGURE 8:**
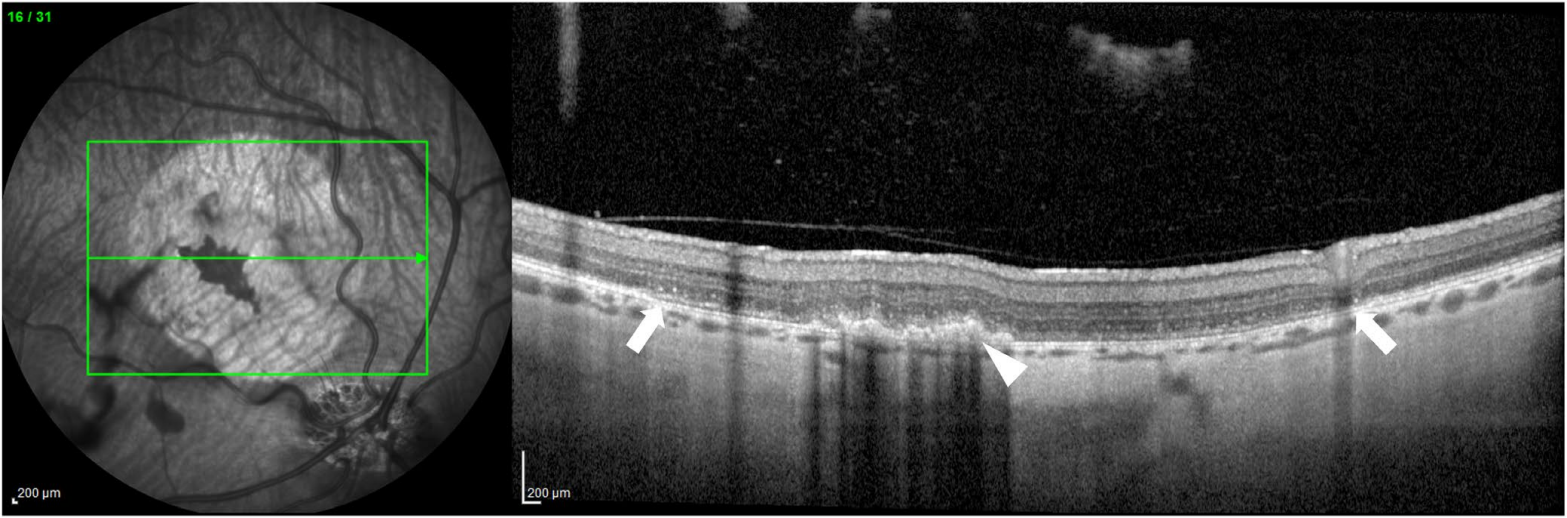
RPE detachment in the minipig. IR cSLO (left panel) and SD‐OCT (right panel) images taken 7 days following subretinal injection in a minipig eye. The small 10–20 μL subretinal bleb showed evidence of diffuse RPE detachment during bleb initiation, so the cannula was repositioned and a second subretinal bleb was created in a different quadrant. Note the accumulation of subretinal material within the central bleb that is hyporeflective on the cSLO image and hyper‐reflective on SD‐OCT (white arrowhead), likely representing accumulation of detached RPE. The surrounding bleb region is hyper‐reflective on cSLO, with thinning of the hyper‐reflective RPE layer on SD‐OCT (white arrows demonstrating bleb margins).

Due to the excessively muscular eyelids of pigs, a locking eyelid speculum can be very valuable to increase exposure. Head positioning, with the nose pointed upward, is also very important to ensure a more central globe positioning to ease the approach. RPE detachment is a common complication during subretinal injection in pigs; this can most often be visualized at bleb induction (Videos 5 and 6). If this occurs, placement of the injection cannula tip in a different location may avoid further RPE detachment. Postoperatively, regions where RPE detachment occurred often experience RPE “scrolling,” giving the reattached retina within the dosing site a striped appearance, with regions of RPE layer thickening separated by zones of absent RPE on optical coherence tomography images (Figure 8). Similar to cats, multiple smaller subretinal blebs of <100 μL each is preferable to a single subretinal bleb of >100 μL, to avoid folding of the retina following reattachment. The authors have routinely performed multiple 50–75 μL injections in pigs without significant retinal folding or prolonged detachment. Due to the inferotemporal orientation of the optic disc, injections are most easily performed nasal and superior to the disc.

**VIDEO 5:** Subretinal injection in the minipig. The fixed‐tip subretinal injection cannula can be visualized within the mid‐vitreous from a superior approach, the optic nerve is along the left margin of the field of view. The cannula tip was cut at an angle prior to insertion into the eye, the open bevel is facing away from the surgeon. As the cannula tip is advanced toward the retina, a shadow of the cannula can be visualized on the retina. The cannula tip is slowly advanced between the nasal retinal vasculature until the shadow meets the tip and slight indentation of the retina is visualized. Infusion is briefly initiated, but retinal detachment does not occur and focal alteration of the RPE is visualized, so the infusion is stopped. The cannula is retracted and advanced once again onto the same retinotomy site, and infusion is initiated once again. Gradual formation of a subretinal bleb is observed in a semicircular shape with focal RPE detachment at the retinotomy but not within the rest of the subretinal bleb. Infusion is continued, and the subretinal bleb slowly expands toward the optic disc and along the vasculature.

**VIDEO 6:** RPE detachment in the minipig. The fixed‐tip subretinal injection cannula is placed along the superior retinal vasculature from a superior approach, two 75 μL subretinal blebs were created for this study. Minimal RPE pigment is visualized sticking to the outer retina during bleb filling, particularly within the inferior aspect of the bleb. Upon initiation of the second subretinal bleb within the nasal quadrant, focal RPE detachment and peeling can be observed near the retinotomy site, followed by diffuse RPE detachment within the bleb, with pigmented RPE clearly still adherent to the outer retina. If this is observed during bleb initiation, infusion should be paused, and the subretinal cannula tip should be retracted and placed in a different site to initiate a proper subretinal injection, if possible.

#### 5.2.5 Non‐human primates

Subretinal injections in non‐human primates are technically easier to perform than in other large animal species, despite the smaller globe volume. This is due to the lower lens:globe volume ratio and proportionally greater vitreous chamber depth, allowing more freedom for manipulation of instruments within the vitreous. The main complication to consider is development of a macular hole during the injection or within the period of reattachment 24–48 h post‐injection, this occurs when the fovea is included in the bleb. Placement of the cannula tip at least two optic disc diameters away from the fovea and ensuring a slow bleb fill rate will minimize risk of macular hole development during the injection. To achieve a submacular injection, which is often the goal of most investigational drug products delivered via subretinal injection, the author prefers to place the cannula tip along the distal vascular arcades temporal to the fovea, but offset from the temporal raphe/horizontal midline (Video 7). Standard subretinal injection volumes in macaques are up to 100 μL for a single bleb, with as much as 150 μL possible in a normal retina without significant complications. A higher prevalence of RPE pigment alteration, persistent detachment within limited portions of the bleb, and subretinal cellular debris can be expected with the higher 150 μL dose volume.

**VIDEO 7:** Subretinal injection in the non‐human primate. The fixed‐tip subretinal injection cannula can be visualized within the mid‐vitreous from a temporal approach, the fovea is at the upper margin of the field of view. The cannula tip was cut at an angle prior to insertion into the eye, the open bevel is facing the fovea. As the cannula tip is advanced toward the retina, a shadow of the cannula can be visualized on the retina. The cannula tip is slowly advanced within the region of the distal temporal vascular arcades, avoiding the midline temporal raphe. Once the shadow meets the tip and slight indentation of the retina is visualized, infusion is initiated using the viscous fluid injection function of a vitrectomy unit. Gradual formation of a subretinal bleb is observed in a semicircular shape, and the subretinal bleb slowly expands and detaches the fovea to achieve the intended submacular delivery of the injected solution.

#### 5.2.6 Sheep

Proof of concept gene therapy for a spontaneously occurring sheep model of achromatopsia has been reported.^38^ As they developed their injection technique, the team started with a three‐port vitrectomy prior to the subretinal injection. However, that resulted in complications, so they settled on a simpler approach without vitrectomy. They placed a 20 g scleral port through the ventral pars plana and used a long flexible IVF‐micropipette for a transvitreal approach to deliver the viral vector into the subretinal space. They found it was safe to deliver up to 600 μL of vector in the adult sheep. In a follow‐on study, the approach was slightly modified; a single 23 g pars plana port was placed 2 mm from the ventral limbus and a 41 g retractable subretinal injection needle (DORC 1270.ext, Dutch Ophthalmic Research Center) was used.^39^

## 6 CONCLUSION

Subretinal injections in animal models require familiarity with the anatomy of the species used, best surgical approach, and potential complications. Above all, sufficient practice is required to ensure adequate reproducibility and minimize procedure‐related effects that may confound efficacy or safety evaluations.

## AUTHOR CONTRIBUTIONS

**Ryan F. Boyd:** Conceptualization (equal); writing—original draft (equal); writing—review and editing (equal). **Simon M. Petersen‐Jones**: Conceptualization (equal); writing—original draft (equal)—review and editing (equal).

## FUNDING INFORMATION

SMP‐J is supported by the Myers‐Dunlap Endowment for Canine Health.

## CONFLICT OF INTEREST STATEMENT

The authors declare no conflicts of interest.

## ETHICAL STATEMENT

This study complies with the ARVO Statement for the Use of Animals in Ophthalmic and Vision Research and was approved by the IACUC of Charles River Laboratories and Michigan State University.

## Data Availability

N/A.
